# Cost-effectiveness of short-term parent-infant-psychotherapy, results from two randomized controlled trials

**DOI:** 10.1186/s12962-025-00696-8

**Published:** 2025-12-23

**Authors:** Benjamin Kaß, Anne Berghöfer, Lars Kuchinke, Christiane Ludwig-Koerner, Franziska Schlensog-Schuster, Stephanie Roll, Thomas Keil, Thomas Reinhold

**Affiliations:** 1 Institute of Social Medicine, Epidemiology and Health Economics, Charité – Universitätsmedizin Berlin, Corporate Member of Freie Universität Berlin and Humboldt-Universität zu Berlin, Luisenstr. 57, 10117 Berlin, Germany; 2 International Psychoanalytic University, Stromstr. 1, 10555 Berlin, Germany; 3 Department of Child and Adolescent Psychiatry, Psychotherapy and Psychosomatics, University of Leipzig, Liebigstr. 20A, 04103 Leipzig, Germany; 4 University Hospital of Child and Adolescent Psychiatry and Psychotherapy and Psychotherapy, University of Bern, Bern, Switzerland; 5 Institute of Clinical Epidemiology and Biometry, University of Würzburg, Josef-Schneider-Str. 2, 97080 Würzburg, Germany; 6 Bavarian Health and Food Safety Authority, State Institute of Health I, 97688 Bad Kissingen, Germany; 7 Institute for Complementary and Integrative Medicine, University Hospital Zurich and University of Zurich, Sonneggstr. 6, Zürich, 8091 Switzerland

**Keywords:** Cost-effectiveness, Parent-infant-psychotherapy, Postpartum, Maternal mental health, Maternal sensitivity

## Abstract

**Background:**

Maternal sensitivity is a crucial factor in fostering infants’ resilience and healthy development. Parent-Infant Psychotherapy (PIP) interventions focus on the improvement of maternal or paternal sensitivity, among other outcomes. Little is known about their cost-effectiveness. This study aimed to evaluate the cost-effectiveness of a short-term PIP compared to care as usual (CAU) in Germany.

**Methods:**

Cost-effectiveness analyses were conducted in two randomized controlled trials (RCTs) of the SKKIPPI project, which investigated the efficacy of a six-week PIP program compared to CAU. PIP aimed to support the establishment and maintenance of healthy parent-infant relationships. The first RCT (RCT-M) focused on mothers with postpartum mental disorders and their infants, while the second (RCT-I) examined children aged 0–36 months with regulatory problems and their mothers. Maternal sensitivity was assessed using the sensitivity subscale of the Emotional Availability Scale (EAS) at baseline, six weeks (primary endpoint), and twelve months, with scores ranging from 1 to 7 (Likert Scale) and higher values showing better sensitivity. Group differences were evaluated using ANCOVAs, adjusted for baseline values and treatment settings (inpatient/outpatient). Health care resource utilization was self-reported via questionnaires at baseline and after twelve months. Costs were estimated using standardized unit costs, and gamma-distributed generalized linear models with log link functions were applied to evaluate cost differences between groups from enrollment to twelve months from a payer’s perspective. Incremental Cost-Effectiveness Ratio (ICER) was calculated if applicable.

**Results:**

Between 2019 and 2021, 51 participants (25 PIP) of RCT-M, and 70 participants (38 PIP) of RCT-I could be included in our analyses. In RCT-M, adjusted EAS scores were slightly lower in the PIP group after twelve months (Δ -0.22, 95% CI -0.55 to 0.11), with higher adjusted costs in the PIP group (Δ €5,603). In RCT-I, the PIP group showed slightly higher adjusted EAS scores (Δ 0.12, 95% CI -0.13 to 0.36), resulting in an ICER of €29,600 per EAS unit gained. Results remained robust in sensitivity analyses.

**Conclusion:**

Cost-effectiveness of the evaluated PIP in improving maternal sensitivity was unlikely in both trials. Future research could focus on mothers with more severe maternal sensitivity problems and alternative effectiveness measures.

**Trial registration:**

German Register for Clinical Trials, ID: DRKS00016353 (RCT-M) and ID: DRKS00017008 (RCT-I).

**Supplementary Information:**

The online version contains supplementary material available at 10.1186/s12962-025-00696-8.

## Background

Postpartum mental health disorders can threaten the foundation of a secure attachment between mothers and their newborns, potentially resulting in negative long-term effects on the child’s and family well-being [1, 2]. Moreover, infants can face regulatory disorders (e.g. excessive crying, sleeping and feeding disorders) which are present in about 20% of all infants and toddlers [3]. Even though the majority of regulatory disorders are transient, they can negatively affect the parent-child-relationship, e.g. due to high level of parental stress and parental burden [4–6].

Maternal sensitivity is important to develop stable and supportive relationships during childhood to establish infant’s resilience and healthy development. Postpartum mental disorders affect maternal sensitivity in responding to the infant [7–9]. Parent-Infant-Psychotherapy (PIP) is a dyadic or triadic intervention that focuses on the establishment of stable relationship patterns, the promotion of the child’s secure attachment, and improvement of maternal or paternal sensitivity [10–12]. In a meta-analysis PIP has been shown to be an effective approach to improve attachment security [12]. Furthermore, the included studies indicated effectiveness with respect to improving maternal sensitivity in at risk populations. However, the authors highlighted the low quality and heterogeneity of included studies and concluded that these limitations should be addressed in further research.

To the best of our knowledge, no cost-effectiveness analyses are available for PIP interventions. Interpersonal therapy of the mother alone has been estimated to result in incremental cost-effectiveness of 1.89 quality-adjusted life-years (QALY) per woman or $8642 per QALY gained compared to routine care when only maternal health system costs were considered [13]. In a more general approach, a recent systematic review investigated the cost-effectiveness of 39 mental health interventions related to antenatal, perinatal, and postnatal mental health conditions [14]. The interventions were heterogeneous regarding intervention components, time frames, and targeted condition. Despite highlighting the lack of sufficient evidence, especially with respect to children, the authors found promising results regarding the cost-effectiveness of investigated programs. The majority of included studies reported cost savings (6 studies), cost-effectiveness [11] or likeliness of cost-effectiveness [7]. Considering the rather short time horizons of the included studies and the identified systematical exclusion of costly long-term consequences on offspring into account, the authors conclude that cost-effectiveness is underestimated. However, none of the included studies investigated PIP.

Maternal mental health burden also has implications for the utilization and costs of health care in the postpartum period. In a recent observational study of the SKKIPPI project, findings demonstrated the importance of sufficient appreciation and treatment of mental health burdens in mothers in the postnatal period [15]. Among others, this study indicated that mothers with noticeable mental health burdens had on average 1713€ higher total costs (compared to those without noticeable mental health burdens) over a mean observation period of 17.5 months since birth.

The aim of our analyses was to investigate the cost-effectiveness of short-term PIP interventions compared to care as usual (CAU) in Germany.

## Methods

We performed cost-effectiveness analyses in two randomized controlled trials of the SKKIPPI project. The SKKIPPI project consists of an observational, population-based cohort study and two RCTs [16]. One of the RCTs focuses on mothers with postpartum mental disorders and their 0–12 months old infants (RCT-M), while the other RCT examined infants and young children with at least one diagnosed regulatory disorder, aged 0–36 months and their mothers (RCT-I). Since the SKKIPPI project in general as well as the two RCTs and the focused PIP intervention in particular have been described elsewhere in detail [6, 16, 17], only a brief description of the intervention is given here.

### Intervention

The focused PIP intervention, used in both RCTs, is based on a short-term focus-based psychodynamic psychotherapeutic intervention manual designed by SKKIPPI core staff members and is oriented on treating children together with their primary care givers, which is traditionally the mother [18]. The intervention aimed to support the building and maintenance of parent-infant-relationships by supporting parents’ ability to mentalize and understand the infant’s affective states. The intervention comprised 12 sequential sessions of 50 min in 6 weeks and was exclusively executed by trained and supervised psychodynamic psychotherapists and psychologists. Therapists in the RCT-I did not work exclusively with the mother and child, but also with the father or other important care givers if necessary. Participants in the CAU groups could receive any standard pedagogical and therapeutic care provided by any suitable source within the German health system other than the PIP intervention over the 6 weeks intervention period.

PIP intervention and CAU took place in either inpatient or outpatient settings. Therefore, the decision about the setting was made upon the severity of the mothers’ or infants’ condition as well as her preferences regarding treatment if possible. Within the inpatient setting, participants of the intervention groups received PIP in addition to standard inpatient psychiatric care.

### Participants

Recruitment of participants of the RCT-M was conducted between January 2019 and December 2021 and between March 2019 and November 2021 in the RCT-I respectively. Recruitment took place in various German study centers located in Berlin, Leipzig, Hamburg, Flensburg, and Potsdam. Randomization in both RCTs took place after an initial decision regarding inpatient or outpatient treatment was made.

Mothers were eligible for study participation in the RCT-M if they were (a) German-speaking, (b) with an ICD-10 diagnose of a mental disorder in the postpartum-period, and (c) their infants were under 12 months of age. Exclusion criteria comprised (a) maternal ICD-10 diagnosis of schizophrenia, substance abuse or recent suicidal ideation, (b) symptoms of alcohol embryopathy or severe chronic organic disease in the child, and (c) participation in further clinical trials or undergoing psychotherapy.

For participation in the RCT-I, infants were eligible if they were (a) aged 36 months or younger, and (b) received an ICD-10 diagnosis of at least one regulatory disorder (e.g. excessive crying, sleeping and feeding disorders). In addition, their mothers needed to be (c) German-speaking, and (d) in a psychological state allowing participation. Exclusion criteria for both the child and mother of the RCT-I comprised (a) any other ongoing clinical trial participation, or (b) receiving any other form of psychotherapy at the beginning of the study. Furthermore, dyads in the RCT-I were excluded if (c) the child had a suspected alcohol embryopathy, or a severe organic impairment or (d) the mother was dealing with acute substance abuse or had suicidal tendencies.

Randomization of participants was independently carried out by a biometrician. Participants in both groups received a monetary compensation of up to 100€ (25€ each after Baseline and 6 weeks’ follow-up and 50€ after the 12 months follow-up).

Even though fathers could be part of the PIP intervention within the RCT-I, the main focus remained on the mothers. Hence, data were only collected from mothers.

### Effectiveness measures

The primary efficacy outcome of both RCTs was defined as maternal sensitivity at the end of the 6-week intervention assessed by the Sensitivity subscale of the Emotional Availability Scale (EAS) [19]. Sensitivity was scored from fifteen-minute videotaped free play interactions between the mothers and infants by trained coders blind to treatment allocation using a Likert Scale (1 to 7) at baseline, after the 6-week intervention period (T1), and after the 12 months follow-up (T2). EAS scores of 3 or below reflect less optimal caregiver sensitivity marked by problematic behaviors such as inappropriate or poorly attuned responses to the child’s signals. Scores of 5 and above indicate healthy and supportive sensitivity, where the caregiver consistently responds appropriately and attunes to the child’s needs. A score of 4 represents an intermediate level of sensitivity, characterized by some appropriately responsive behaviors alongside lapses that indicate inconsistent or only partially adequate sensitivity.

### Health care utilization and costs

Health care resource utilizations were assessed in both RCTs with a self-developed patient questionnaire at baseline for the period from the birth of the index child to study enrollment and for the period from baseline to T2. Underlying costs of the self-reported utilization data were calculated by applying standardized German unit costs based on 2021 prices to the utilization data [15, 20]. Furthermore, stakeholders from the area of early support services to a nationwide operating health insurance company were involved to value the utilization of often heterogeneous and locally operating early support services. Table 1 gives an overview of the included cost components, its monetary valuation on a visitation or daily basis.

**Table 1:** Cost components, its monetary valuation and sources

Cost components	Monetary Valuation (€) (Visitation or daily costs)	Source
**Intervention**		
PIP session	102.57 (visit)	German unified doctor’s fee scale
Travel expenses (outpatient visits)	10.23 (visit)	Study agreement
**Mother**		
General Practitioner	22.99 (visit)	Bock et al. 2015*
Gynecologist	34.53 (visit)	Bock et al. 2015
Psychotherapy	102.57 (visit)	German unified doctor’s fee scale
Emergency room	428.99 (visit)	Bock et al. 2015
Hospital stays	659.99 (daily costs)	Bock et al. 2015
Rehabilitation care	139.07 (daily costs)	Bock et al. 2015
Addiction counseling [visits]	50 (visit)	Stakeholder**
Index child***		
Pediatrician	39.35 (visit)	Bock et al. 2015
Early Detection Screenings (age dependent)	14.02–44.72 (visit)	German unified doctor’s fee scale
Emergency room	428.99 (visit)	Bock et al. 2015
Hospital stays	659.99 (daily costs)	Bock et al. 2015
Ergotherapy	42.99 (visit)	Bock et al. 2015
Logotherapy	44.22 (visit)	Bock et al. 2015
Osteopathy	85 (visit)	Mean visitation costs of a statutory health insurance company
Physiotherapy	18.82 (visit)	Bock et al. 2015
**Mother & Index child**		
Standard Midwife care [6 weeks utilization]	315.36 (overall program costs)	German Midwifes’ Fee Scale
Additional Midwife care	38.46 (visit)	German Midwifes’ Fee Scale
Mother-Child-Treatment course	139.07 (visit)	Stakeholder
*Early support services*		
Expert consultation (at visitor center)	50 (visit)	Stakeholder
Expert consultation (at home)	65 (visit)	Stakeholder
Accommodation at Mother-Child facility	275 (daily costs)	Stakeholder
Accommodation at Fulltime Care Facility	34.37 (daily costs)	Stakeholder
Crying counseling	50 (visit)	Stakeholder

* valued with 2021 prices

** Stakeholders include workers in the field, medical doctors, local authorities and representatives of institutions for aiding youth, who preferred not to be mentioned by name or institution

*** The child whose birth led to recruitment of the mother-infant dyad to the study

Total health care resource utilization costs at baseline and T2 were calculated from a payer’s perspective. Thereby, we classified the available cost components into costs (a) attributable to the intervention in general (comprising PIP treatment and outpatient travel expenses), (b) only attributable to the mothers (comprising visitation costs for general practitioners, gynecologists, psychiatric treatment, emergency rooms, hospital stays, rehabilitation care and addiction counseling sessions), (c) only attributable to the index children (pediatrician, early detection screenings, emergency rooms, hospital stays, ergotherapy, logotherapy, osteopathy and physiotherapy), (d) attributable to mothers and index children (midwife care, mother-child-treatment courses other than the PIP intervention and early support services (including expert consultation and crying counseling)).

Intervention sessions and general psychotherapy treatment after study enrollment are valued with 102.57€ according to the 2021 German unified doctor’s fee scale [21]. Outpatient therapy home visits are valued in Germany depending on the time of the day of visitation and distance between the medical office and the residence of the patients. For the simplification of payment flows, it was a priori determined between the funder and research group to refund an additional 10.23€ for travel expenses per outpatient visit in the intervention groups. The same value was applied for outpatient psychotherapy treatment in the control groups. Cost valuation was made on a 2021 basis for easier comparability with the cost results of the SKKIPPI cohort study [15]. The participation compensation was not included in the analyses since all participants were compensated (PIP and CAU groups) and these costs are irrelevant for future potential payers.

### Statistical analyses

Sociodemographic baseline characteristics, health care resource utilization costs, EAS sensitivity are displayed as means and standard deviations (SD) or proportions in both RCTs. The statistical analyses are based on the intention-to-treat principle (without imputation of missing values). Only mother-infant-dyads with both available effectiveness and cost data were included in the analyses.

Analyses of covariances (ANCOVAs) were performed to determine differences of EAS sensitivity after 6 weeks and 12 months in both studies. Treatment (PIP vs. CAU) was included in the model as independent variable, and we adjusted for maternal sensitivity at baseline as well as treatment setting (inpatient or outpatient). Results are displayed as adjusted means of the respective treatment group and 95% confidence intervals (CIs).

Taking the skewness of health care cost data in both RCTs into account, gamma distributed generalized linear models with log link function adjusted for health care costs between birth and baseline as well as intervention setting were applied to estimate mean adjusted costs with 95% CIs and cost differences between the respective groups from baseline to T2 [22, 23].

The cost-effectiveness of the short-term PIP compared to CAU were investigated after the 12 months follow-up period in terms of EAS differences and cost differences from a payers perspective. Results are displayed as adjusted average costs per one unit EAS difference gain. As the study duration was limited to 12 months, no discounting of costs or EAS outcomes was applied.

All statistical analyses were conducted with R version 4.2.1 or higher [24].

### Sensitivity analyses

The uncertainty of single cost parameters in the RCTs was investigated by one-way deterministic sensitivity analysis. Thereby, underlying unit costs were consecutively varied within predefined minimum-maximum ranges (± 20% of the base case cost assumption) and plotted in tornado diagrams. Moreover, Monte-Carlo processes with 1000 random iterations were conducted as probabilistic sensitivity analyses on the underlying unit costs of the total cost results after 12 months. Cost components were allowed to vary within the ± 20% ranges of the deterministic sensitivity analyses. In addition, since the studies started prior to and ended in the middle of the SARS-CoV-2 pandemic, we investigated whether a recruitment prior to the first lockdown in Germany impacted the total costs after 12 months. Therefore, we included the binary variable baseline prior to first SARS-CoV-2 lockdown as an additional dependent variable in the gamma distributed generalized linear models with log link function. The beginning of school closing in Berlin on the 16th of March 2020 was defined as the lockdown beginning. Furthermore, given the observed baseline differences in educational level (RCT-M) and in medical complications during pregnancy (RCT-I), we conducted sensitivity analyses to examine their potential influence on total costs at 12 months. These variables were added to the respective models in the same way as previously done to assess the impact of the COVID-19 pandemic.

## Results

A total of 120 mother-infant-dyads were included in the RCT-M (57 PIP group) and 140 in the RCT-I (67 PIP group). EAS (6 weeks and 12 months) and cost data (12 months) were available for 51 (25 PIP group) dyads in the RCT-M and 70 (38 PIP group) dyads in the RCT-I. Baseline characteristics of these participants including mean costs from births of the index children to baseline are displayed in Table 2. The main sociodemographic characteristics between the groups were mostly comparable at baseline in respective RCTs. Differences in the RCT-M were observed in (a) the educational level (80.0% of the participants in the PIP group had a high educational level compared to 69.2% in the CAU group), (b) planned pregnancy with the index child (PIP: 64.0% vs. CAU: 76.9%) and (c) total costs between birth and baseline (PIP: 12,383€ ±24,881€ vs. CAU: 6,811€ ±18,326€). In the RCT-I, main group differences were observed in (a) the distribution of number of children in the household (PIP: 41.4% 2 or more children vs. CAU: 28.2%), (b) medical complications during pregnancy (PIP: 31.6% vs. CAU: 15.6%) and (c) total costs between birth and baseline (PIP: 19,776 €, SD ± 32,241 vs. CAU: 16,044€, SD ± 25,804€).

**Table 2:** Characteristics of the study populations at baseline

	RCT Mothers (RCT-M)		RCT Infants (RCT-I)	
	Parent-Infant-Psychotherapy (PIP), *n* = 25 *n* (%) or mean ± SD	Care as usual (CAU), *n* = 26 *n* (%) or mean ± SD	Parent-Infant-Psychotherapy (PIP), *n* = 38 *n* (%) or mean ± SD	Care as usual (CAU), *n* = 32 *n* (%) or mean ± SD
Age (Mother) in years	34.1 ± 4.0	33.2 ± 3.7	33.4 ± 5.0	34.2 ± 4.4
Educational level (based on ISCED*)				
* Middle*	5 (20.0)	4 (15.4)	9 (23.7)	10 (31.3)
* High*	20 (80.0)	18 (69.2)	22 (57.9)	16 (50.0)
* Unknown*	-	4(15.4)	7 (18.4)	6 (18.8)
Single parent (not living together with second custodial parent)	2 (8.0)	4 (15.4)	4 (10.5)	3 (9.4)
* Unknown*	4 (16.0)	2 (7.7)	8 (21.1)	6 (18.8)
Age (Index child) in months	4.9 ± 3.9	5.2 ± 3.2	17.0 ± 10.0	21.0 ± 11.0
Female (Index child)	13 (52.0)	11 (42.3)	21 (55.3)	15 (46.9)
Number of children < 18 years in the household (including index child)				
* 1*	14 (56.0)	14 (53.8)	12 (31.6)	17 (53.1)
* 2*	2 (8.0)	5 (19.2)	14 (38.8)	6 (18.8)
* 3 or more*	1 (4.0)	4 (15.3)	1 (2.6)	3 (9.4)
* unknown*	8 (32.0)	3 (11.5)	11 (28.9)	6 (18.8)
Preterm delivery	0 (0.0)	3 (11.5)	5 (13.2)	3 (9.4)
* unknown*	8 (32.0)	5 (19.2)	12 (31.6)	13 (40.6)
Planned pregnancy	16 (64.0)	20 (76.9)	23 (60.5)	20 (62.5)
* unknown*	1 (4.0)	2 (7.7)	9 (23.7)	8 (25.0)
Psychological stress during pregnancy	18 (72.0)	19 (73.1)	15 (39.5)	10 (31.3)
* unknown*	5 (20.0)	5 (19.2)	11 (28.9)	8 (25.0)
Medical complications during pregnancy	13 (52.0)	11 (42.3)	12 (31.6)	5 (15.6)
* unknown*	5 (20.0)	7 (26.9)	5 (13.2)	7 (21.9)
Index child diagnosed with serious illness or disability after birth	4 (16.0)	2 (7.7)	13 (34.2)	9 (28.1)
* unknown*	5 (20.0)	4 (15.3)	9 (23.7)	12 (37.5)
Study center				
IPU** Berlin	20 (80.0)	19 (73.1)	10 (26.3)	4 (12.5)
* University hospital Leipzig*	-	-	23 (60.5)	23 (71.9)
* St. Joseph hospital Berlin - Weissensee*	3 (12.0)	4 (15.3)	-	-
* University hospital Hamburg-Eppendorf*	0 (0.0)	1 (3.8)	-	-
* FZ* ^*^ *Potsdam*	2 (8.0)	2 (7.7)	10 (26.3)	4 (12.5)
* Helios Parkclinic Leipzig*	-	-	1 (2.6)	2 (6.2)
* Vivantes Berlin Neukoelln*	-	-	1 (2.6)	1 (3.1)
Outpatient treatment *(study setting)*	22 (88.0)	23 (88.5)	32 (84.2)	24 (75.0)
Baseline prior to first SARS-CoV-2 Lockdown	8 (32.0)	9 (34.6)	18 (47.4)	16 (50.0)
EAS^**^ Sensitivity	3.53 ± 0.48	3.31 ± 0.57	3.34 ± 0.48	3.43 ± 0.62
Costs (from birth to Baseline), €				
* Mother*	5,777 ± 9844	1,896 ± 5,623	3742 ± 9076	3,319 ± 10,702
* Index Child*	5,930 ± 17,879	4,419 ± 15,569	11,612 ± 21,418	12,136 ± 22,718
* Costs associated with mother & index Child (incl. Early Support)*	676 ± 484	497 ± 275	4,421 ± 23,056	589 ± 996
* Total*	12,383 ± 24,881	6,811 ± 18,326	19,776 ± 32,241	16,044 ± 25,804

* ISCED: International standard classification of education

** IPU: International Psychoanalytic University

^*^ FZ: family center

^**^ EAS: Emotional Availability Scale (Likert Scale 1 to 7); ≤ 3 problematic interactions, 4 inconsistent interactions, ≥ 5 healthy interactions

The adjusted and unadjusted mean EAS results on maternal sensitivity from Baseline to 12 months follow-up are provided in Table 3. Maternal sensitivity in both RCTs and both groups were similar and slightly below the threshold of showing problematic behavior in the mother-infant-dyads at baseline in all groups. These results persist over 6 weeks as well as the 12 months follow-up periods.

**Table 3:** Unadjusted and adjusted mean emotional availability scale scores on maternal sensitivity until 12 months follow-up

	RCT Mothers (RCT-M)			RCT Infants (RCT-I)		
	Parent-Infant-Psychotherapy (PIP), *n* = 25	Care as usual (CAU), *n* = 26	Difference*	Parent-Infant-Psychotherapy (PIP), *n* = 38	Care as usual (CAU), *n* = 32	Difference*
**Baseline**						
*unadjusted EAS* ^*^ *mean (± SD)*	3.53 (± 0.48)	3.31 (± 0.57)	0.22	3.34 (± 0.58)	3.43 (± 0.62)	-0.09
***6 weeks***						
*unadjusted EAS* *mean (± SD)*	3.50 (± 0.56)	3.54 (± 0.57)	-0.04	3.32 (± 0.63)	3.43 (± 0.56)	-0.11
adjusted EAS** *mean (95% CI)*	3.46 (3.18–3.74)	3.59 (3.30–3.87)	-0.13 (-0.44-0.18)	3.34 (3.11–3.56)	3.42 (3.19–3.65)	-0.08 (-0.37-0.20)
***12 months***						
*unadjusted EAS* *mean (± SD)*	3.36 (± 0.60)	3.53 (± 0.55)	-0.17	3.48 (± 0.48)	3.34 (± 0.58)	0.14
*adjusted EAS* *mean (95% CI)*	3.34 (3.04–3.64)	3.56 (3.26–3.87)	-0.22 (-0.55-0.11)	3.38 (3.19–3.57)	3.26 (3.07–3.46)	0.12 (-0.13-0.36)

* Emotional Availability Scale (Likert Scale 1 to 7); ≤ 3 low sensitivity, 4 intermediate sensitivity, ≥ 5 adequate sensitivity

* Parent-Infant-Psychotherapy group – Care as usual group

** Adjusted for baseline value and intervention setting (inpatient/outpatient)

Mean adjusted health care costs in both RCTs were higher in the intervention groups compared to CAU (Table 4). Combination of effectiveness and cost results (adjusted for baseline value and intervention setting) after 12 months showed an ICER of 29,600€ per additional unit on the EAS maternal sensitivity scale in the RCT-I. With slightly higher adjusted mean maternal sensitivity scores in the CAU group (Difference − 0.22, 95% CI -0.55 to 0.11) and higher adjusted mean total costs in the PIP group (5,603€) of the RCT-M the ICER was not calculated. Underlying health care resource utilization data are presented in Additional file 1 (RCT-M) & Additional file 2 (RCT-I).

**Table 4:** Unadjusted and adjusted mean health care costs from baseline until 12 months follow-up

	RCT Mothers (RCT-M)			RCT Infants (RCT-I)		
	Parent-Infant-Psychotherapy (PIP), *n* = 25	Care as usual (CAU), *n* = 26	Difference*	Parent-Infant-Psychotherapy (PIP), *n* = 25	Care as usual (CAU), *n* = 26	Difference*
	Mean (€) (SD) or (95%CI)	Mean (€) (SD) or (95%CI)	Δ €	Mean (€) (SD) or (95%CI)	Mean (€) (SD) or (95%CI)	Δ €
***Unadjusted costs***						
*Intervention*	1,151 (± 447)	-	-	1,106 (± 387)	-	-
*Mother*	1,935 (± 2,119)	3,795 (± 11,952)	-1,860	1,980 (± 6,216)	2,668 (± 7,159)	-688
*Index Child*	3,405 (± 7,596)	2,062 (± 3,997)	1,343	4,673 (± 10,757)	3,718 (± 8,077)	955
*Costs associated with mother & index child [including Early Support]*	746 (± 916)	569 (± 987)	177	385 (± 890)	1,059 (± 2,646)	-674
***Total***	7,236 (± 7,889)	6,424 (± 15,990)	812	8,144 (± 12,111)	7,445 (± 15,171)	699
***Adjusted costs***						
***Total*** *[baseline costs and intervention setting (inpatient/outpatient)]*	13,522 (7,509 − 24,347)	7,919 (4,406 − 14,232)	5,603	11,664 (6,701 − 20,300)	8,112 (4,708 − 13,977)	3,552

* Parent-Infant-Psychotherapy group – Care as usual group

**Fig. 1:**
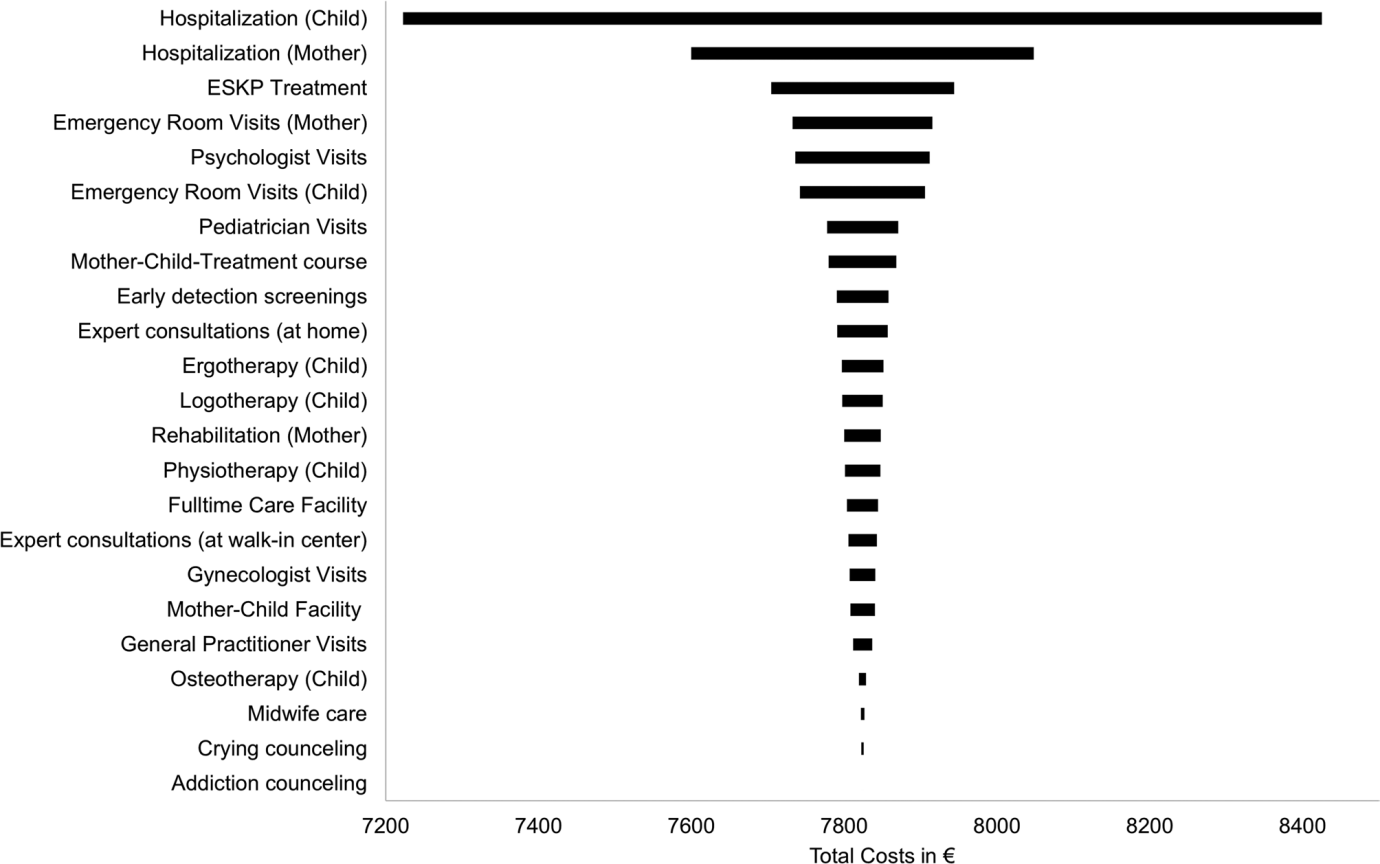
Tornado diagram of impact of consecutive ± 20% variation in unit costs on total costs (RCT-M)

**Fig. 2:**
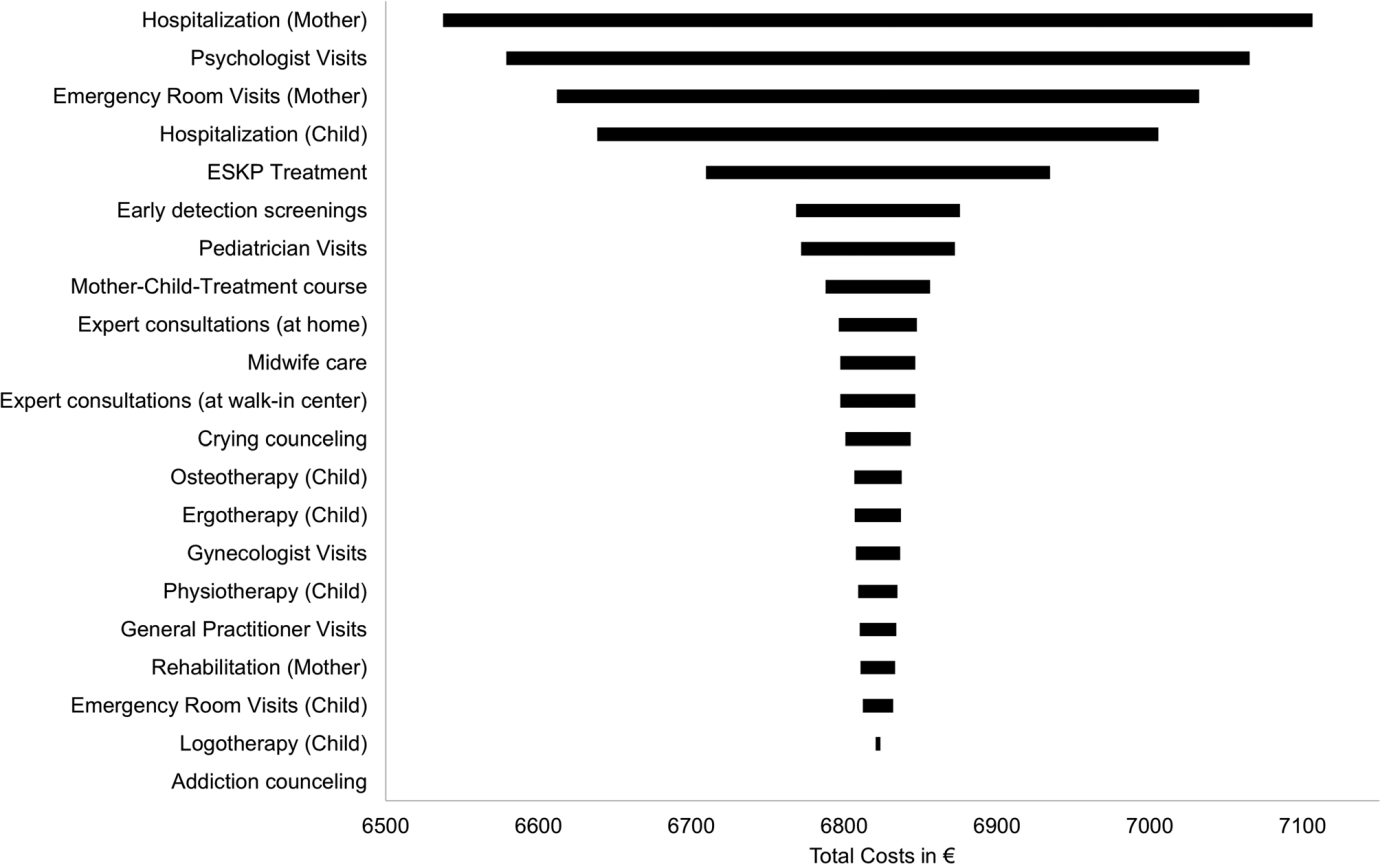
Tornado diagram of impact of consecutive ± 20% variation in unit costs on total costs (RCT-I)

The cost results were robust to all sensitivity analyses. The deterministic sensitivity analyses showed that changes in the valuation of hospitalizations of the mothers had the largest impact on mean total costs in both RCTs. Moreover, the variation of PIP treatment costs showed some impact on mean total costs in both RCTs (Figs. 1 and 2). Similar mean total cost differences between the PIP and CAU groups were observed in the Monte Carlo simulations. The total cost difference (PIP-CAU group) in the RCT-M was 827€ (Monte Carlo simulation) compared to 812€ (base case) and 709€ (Monte Carlo simulation) compared to 699€ (base case) in the RCT-I. Furthermore, the inclusion of the binary lockdown variable to the cost model did not change the overall direction of the mean adjusted cost results (adjusted mean total cost differences PIP-CAU after 12 months: RCT-M: 5,697€; RCT-I: 3,543€). Additionally, the adjusted mean total cost differences remained materially unchanged when accounting for baseline differences in educational level in RCT-M and medical complications during pregnancy in RCT-I (RCT-M: €6,585; RCT-I: €3,412).

## Discussion

In this study we performed cost-effectiveness analyses alongside two randomized controlled trials of the SKKIPPI project to investigate the cost-effectiveness of a short-term PIP intervention compared to care as usual. The following findings reflect the main results of our analyses: First, despite promising results in previous studies, cost-effectiveness of the investigated short-term PIP is unlikely in both investigated RCTs with respect to maternal sensitivity. Second, the maternal sensitivity of the study populations barely changed in all groups over the observational period of the study and did not show inappropriately low sensitivity (EAS ≤ 3) on average in any group. Even though the scores were on average below the intermediate level of sensitivity (EAS = 4), it can be argued that the randomly selected study populations might have been too healthy or of overall inconsistent maternal sensitivity to detect relevant changes on the maternal sensitivity subscale of the EAS. Third, the COVID-19 pandemic had an enormous impact on both studies with respect to study recruitment and execution. Hence, both RCTs did not reach the targeted study populations of 180 mother-infant-dyads. Additionally, the execution of the intervention was hindered by the mandatory wearing of respiratory masks. Mask-wearing potentially harmed the mother-child and therapist-dyad interactions as well as the evaluation of the video-documented mother-child interaction to assess maternal sensitivity. Lastly, the available data lacked completeness in numerous cases and only participants with available cost and EAS data were evaluated.

The effectiveness results of the investigated RCTs are in line with the literature, indicating no superiority of the short-term PIP intervention in comparison to other treatment options [12]. However, the overall low effects on the EAS scales over the 12-month follow-up periods in both RCTs should be discussed with respect to the validity of this assessment in relation to the study objectives. The high initial values and the associated good emotional availability of the mothers in both RCTs at the beginning of the studies indicate that the assessment mother-child play interactions, although representing the current research standard, is not ideally suited for detecting differences in emotional availability within these clinical samples. Future research could aim to increase the validity of emotional availability assessments by basing EAS measurements on more conflict-ridden interaction situations and targeting higher-risk populations with lower levels of maternal sensitivity at baseline. Furthermore, the necessity of employing alternative measurement instruments and objectives to evaluate the effectiveness of PIP must be deliberated. Such instruments may include additional scales of the EAS, such as hostility, the stress experience of parents [25] or child psychopathology [26].

Taking our recent findings regarding the importance of sufficient appreciation and treatment of mental health burdens in mothers in the postnatal period into account [15], it can be concluded that despite the SARS-CoV 2 pandemic the mother-infant-dyads in both studies and all groups received on average sufficient treatment to at least stabilize maternal sensitivity. Therefore, the new more intense PIP treatment was more expensive than regular CAU treatment.

### Strengths and limitations

To the best of our knowledge, no cost-effectiveness analyses of PIP interventions have been conducted to this point. Our analyses of two PIP RCTs with slightly different target groups are therefore hopefully a steppingstone for further high-quality cost-effectiveness analyses in the field of PIP interventions.

Our findings are limited by the self-reported character of health care utilization and the associated uncertainties regarding the participants’ responses, e.g. in form of potentially untruthful replies and recall bias. Moreover, completeness of data in combination with recruitment problems were major concerns in both studies, resulting in a potentially smaller and different sample than initially intended. Furthermore, we could only consider variable costs of the intervention (PIP sessions and transportation costs) and had to leave out the implementation and training costs. However, this approach is justifiable, because such implementation costs per patient are in any case subject to large economies of scale, which become increasingly negligible with an increasing spread of an intervention. Additionally, we assumed that psychotherapy was held in one-on-one sessions, and we have not considered potential discounts for group therapy sessions. The effect of these challenges on overall costs remains unclear. However, all total cost findings were robust to sensitivity analyses.

Participants in both RCTs were highly educated and predominantly of German heritage which challenges the generalizability of our results to the general population. The focus on mother-infant-dyads, which was justified in both RCTs with the assumption of mothers being the predominant reference persons of the children after birth, ignores the potential PIP impact on fathers, which further undermines the generalizability of our findings.

The RCTs were conducted in a multicenter setting. Although the implementation of PIP was specified by a manual and should only be carried out by approved therapists, it remains uncertain whether all treatments were actually carried out in a sufficiently standardized manner at all times.

Moreover, no clinically important minimum difference of EAS subscale on maternal sensitivity is defined. Hence, our interpretations of maternal sensitivity results are based on clinical advisers.

## Conclusions

The evaluated short-term, focused-based Parent-Infant-Psychotherapy was not cost-effective compared to care as usual. This is mainly based on the similar maternal sensitivity in all groups, in combination with additional health care costs in the intervention groups from a payers’ perspective. The COVID-19 pandemic, with burdens such as mandatory mask wearing, affected conduct of the studies and likely influenced the results. Based on our experiences, future research in the field could focus on mothers with more severe maternal sensitivity problems and include alternative, more sensitive effectiveness measures e.g. infant psychopathology or attachment to investigate the effects of Parent-Infant-Psychotherapy interventions.

## Supplementary Information

Below is the link to the electronic supplementary material.

Supplementary Material 1

Supplementary Material 2

## Data Availability

The datasets used and/or analysed during the current study are available from the corresponding author on reasonable request.

## Abbreviations

CAU: Care As Usual
CI: Confidence Interval
EAS: Emotional Availability Scale
PIP: Parent-Infant-Psychotherapy
RCT: Randomized Controlled Trial
SD: Standard Deviation
