# Supplementary material for: Cost-effectiveness of short-term parent-infant-psychotherapy, results from two randomized controlled trials
Source: Cost Eff Resour Alloc. 2025 Dec 23;24:13. doi: 10.1186/s12962-025-00696-8 (PMC12837010; doi:10.1186/s12962-025-00696-8)
Supplement: Supplementary file 1 — Supplementary Material 1 [file 12962_2025_696_MOESM1_ESM.pdf]

Additional file 1. Utilization of healthcare services from baseline to 12 months follow-up (RCT-M)

	Parent-Infant-Psychotherapy (PIP), n=25					Care as usual (CAU), n=26				
		all participants		participants with at least one visit/day of stay			all participants		participants with at least one visit/day of stay	
	n† (%)	Mean (SD)	Median (IQR)	Mean (SD)	Median (IQR)	n† (%)	Mean (SD)	Median (IQR)	Mean (SD)	Median (IQR)
**Intervention [visits]**	22 (88)	10.28 (4.0)	12 (0)	11.68 (1.0)	12 (0)	0 (0)	-	-	-	-
**Mother**										
General Practitioner [visits]	20 (80)	2.6 (2.4)	2 (4)	3.3 (2.3)	2.5 (3.3)	21 (81)	2.7 (3)	2 (2)	3.3 (3)	3 (2)
Gynecologist [visits]	21 (84)	2.1 (2.4)	1 (1)	2.5 (2.5)	2 (2.0)	23 (88)	2.2 (1.8)	2 (1,75)	2.4 (1.7)	2 (2)
Psychiatric treatment [visits]	18 (72)	12.4 (17.2)	8 (20)	17.2 (18.2)	10 (16.5)	23 (88)	11.4 (11.2)	8,5 (12,5)	12.9 (11.1)	10 (13.5)
Emergency room [visits]	3 (12)	0.2 (0.6)	0 (0)	1.7 (0.6)	2 (0.5)	1 (4)	0 (0.2)	0 (0)	1 (-)	-
Hospital stays [days]	4 (16)	0.7 (1.7)	0 (0)	4.3 (1.3)	4 (0.8)	2 (8)	3.6 (17.6)	0 (0)	46.5 (61.5)	46.5 (43.5)
Rehabilitation care [days]	0 (0)	-	-	-	-	1 (4)	0.8 (4.1)	0 (0)	21 (-)	-
Addiction counseling [visits], N=50	0 (0)	-	-	-	-	0 (0)	-	-	-	-
**Index child**										
Pediatrician [visits]	25 (100)	6.9 (3.7)	7 (6)	6.9 (3.7)	7 (6)	26 (100)	5.9 (3.4)	6 (2,75)	5.9 (3.4)	6 (2.8)
Early Detection Screenings [visits], N=50	24 (96)	6.7 (0.9)	7 (0)	6.7 (0.9)	7 (0)	26 (100)	6.9 (0.3)	7 (0)	6.9 (0.3)	7 (0)
Emergency room [visits]	12 (48)	4.4 (17.9)	0 (1)	9.3 (25.5)	1 (1.8)	10 (38)	0.5 (0.8)	0 (1)	1.4 (0.7)	1 (0.8)
Hospital stays [days], N=37	8 (32)	1.4 (1.9)	0 (2)	3.3 (1.6)	2.5 (2.3)	4 (15)	2.5 (7.2)	0 (0)	11.3 (12.8)	6 (11.3)
Ergotherapy [visits]	3 (12)	2.8 (12.1)	0 (0)	23.7 (31.8)	10 (29.5)	1 (4)	0.8 (3.9)	0 (0)	20 (-)	-
Logotherapy [visits]	1 (4)	0.4 (2)	0 (0)	10 (-)	-	0 (0)	-	-	-	-
Osteopathy [visits]	10 (40)	1 (1.5)	0 (2)	2.4 (1.3)	2 (1.8)	7 (27)	0.9 (1.8)	0 (0,75)	3.3 (2)	3 (2.5)
Physiotherapy [visits]	3 (12)	3.3 (10.8)	0 (0)	27.3 (20)	20 (19)	6 (23)	3.7 (9)	0 (0)	15.8 (13.3)	12 (7.5)
**Mother & Index child**										
Additional Midwife care [visits]	9 (36)	5.3 (10.2)	0 (4)	14.7 (12.5)	10 (16)	2 (8)	1.2 (5.9)	0 (0)	16 (19.8)	16 (14)
Mother-Child-Treatment course [days]	0 (0)	0 (0)	0 (0)	0 (0)	0 (0)	3 (12)	2.4 (6.8)	0 (0)	21 (0)	21 (0)
Early support services										
Expert consultation (at walk-in center) [visits], N=50	11 (44)	2.8 (4.8)	0 (3)	6.3 (5.6)	4 (7)	8 (31)	2.3 (4.8)	0 (1)	7.1 (6.3)	5 (8.5)

Expert consultation (at home) [visits], N=50	8 (32)	3.4 (7.4)	0 (1)	10.6 (10.1)	10 (10)	4 (15)	0.6 (2.1)	0 (0)	4 (4.2)	2,5 (4.5)
Accommodation at Mother-Child Facility [days]	0 (0)	-	-	-	-	0 (0)	-	-	-	-
Accommodation at Fulltime Care Facility [days]	0 (0)	-	-	-	-	0 (0)	-	-	-	-
Crying Counseling [visits], N=50	9 (36)	3.7 (6.9)	0 (6)	3.3 (2.3)	2.5 (3.3)	3 (12)	0.7 (2.2)	0 (0)	6 (3.6)	5 (3.5)

† Any visit or utilization

*binary question if standard midwife care was utilized
