# Supplementary material for: Cost-effectiveness of short-term parent-infant-psychotherapy, results from two randomized controlled trials
Source: Cost Eff Resour Alloc. 2025 Dec 23;24:13. doi: 10.1186/s12962-025-00696-8 (PMC12837010; doi:10.1186/s12962-025-00696-8)
Supplement: Supplementary file 2 — Supplementary Material 2 [file 12962_2025_696_MOESM2_ESM.pdf]

Additional file 2. Utilization of healthcare services from baseline to 12 months follow-up (RCT-I)

	Parent-Infant-Psychotherapy (PIP), n=25					Care as usual (CAU), n=26				
		all participants		participants with at least one visit/day of stay			all participants		participants with at least one visit/day of stay	
	n† (%)	Mean (SD)	Median (IQR)	Mean (SD)	Median (IQR)	n† (%)	Mean (SD)	Median (IQR)	Mean (SD)	Median (IQR)
**Intervention [visits]**	34 (90)	10.7 (3.7)	12 (0)	11.9 (0.3)	12 (0)	0 (0)	-	-	-	-
**Mother**										
General Practitioner [visits]	31 (82)	3.1 (3)	2 (3.8)	3.8 (2.8)	3 (3)	24 (75)	2.4 (2.5)	2 (3.3)	3.3 (2.4)	2.5 (2)
Gynecologist [visits]	30 (79)	2.5 (3.2)	1 (1.8)	3.1 (3.3)	2 (2.8)	21 (66)	2.5 (4.8)	1 (2)	3.9 (5.5)	2 (1)
Psychiatric treatment [visits]	17 (45)	4.9 (9.7)	0 (5)	11 (12.2)	5 (12)	14 (44)	3.6 (7)	0 (2.8)	8.1 (8.7)	5.5 (8)
Emergency room [visits]	4 (11)	1.8 (10.5)	0 (0)	17.5 (31.7)	2 (17.5)	4 (13)	0.2 (0.4)	0 (0)	1.3 (0.5)	1 (0.3)
Hospital stays [days]	4 (11)	0.6 (2)	0 (0)	5.5 (3.7)	5 (5)	7 (22)	3 (10.8)	0 (0)	13.9 (20.7)	6 (6.5)
Rehabilitation care [days]	1 (3)	1.1 (6.5)	0 (0)	40 (-)	-	1 (3)	0.7 (3.7)	0 (0)	21 (-)	-
Addiction counseling [visits]	0 (0)	-	-	-	-	0 (0)	-	-	-	-
**Index child**										
Pediatrician [visits]	38 (100)	6.9 (6.9)	5 (6.5)	6.9 (6.9)	5 (6.5)	28 (88)	4.8 (4.3)	4 (4)	5.5 (4.1)	4 (4.3)
Early Detection Screenings [visits], N=40	25 (66)	7.4 (0.5)	7 (1)	7.4 (0.5)	7 (1)	15 (47)	7.3 (0.6)	7 (1)	7.3 (0.6)	7 (1)
Emergency room [visits]	15 (39)	1 (1.9)	0 (1)	2.6 (2.3)	2 (2)	12 (38)	0.9 (1.5)	0 (1)	2.3 (1.7)	2 (2)
Hospital stays [days], N=62	13 (34)	6.5 (17.4)	0 (2)	14.5 (24.2)	3 (8)	6 (19)	5.7 (14)	0 (3)	21.7 (21.1)	13.5 (20.5)
Ergotherapy [visits]	8 (21)	4.2 (10.3)	0 (0)	19.8 (14.4)	22.5 (20.3)	6 (19)	2.1 (5)	0 (0)	11 (6.3)	11.5 (7.8)
Logotherapy [visits]	9 (24)	3.3 (8.3)	0 (0)	13.9 (12.4)	10 (15)	6 (19)	2.7 (9.5)	0 (0)	14.5 (18.8)	7 (15)
Osteopathy [visits]	8 (21)	0.4 (0.8)	0 (0)	1.8 (0.7)	2 (1)	4 (13)	0.2 (0.5)	0 (0)	1.5 (0.6)	1.5 (1)
Physiotherapy [visits], N=69	13 (34)	6.6 (12.9)	0 (6.8)	19.2 (15.7)	10 (23)	8 (25)	6 (13.1)	0 (3)	23.3 (16.5)	20 (22.5)
**Mother & Index child**										
Additional Midwife care [visits]	3 (8)	0.7 (2.7)	0 (0)	8.3 (6.4)	12 (5.5)	0 (0)	-	-	-	-
Mother-Child-Treatment course [days]	2 (5)	1.3 (5.6)	0 (0)	24.5 (4.9)	24.5 (3.5)	3 (9)	2 (6.2)	0 (0)	21 (0)	21 (0)
Early support services										
Expert consultation (at walk-in center) [visits], N=68	10 (26)	0.9 (2)	0 (1)	3.3 (2.7)	3 (2.5)	9 (28)	0 (1)	11 (15.5)	8 (8)	3.2 (9.5)

Expert consultation (at home) [visits], N=69	4 (11)	1.9 (7)	0 (0)	17.5 (15.1)	11 (9.5)	2 (6)	0 (0)	55 (7.1)	55 (5)	3.4 (13.6)
Accommodation at Mother-Child Facility [days]	0 (0)	-	-	-	-	1 (3)	0.1 (0.5)		3 (-)	-
Accommodation at Fulltime Care Facility [days]	0 (0)	-	-	-	-	1 (3)	1.0 (5.4)		30 (-)	-
Crying Counseling [visits]	3 (8)	0.3 (1.4)	0 (0)	4.3 (3.2)	3 (3)	0 (0)	-	-	-	-

† Any visit or utilization

*binary question if standard midwife care was utilized
